# Evaluation of bumetanide as a potential therapeutic agent for Alzheimer’s disease

**DOI:** 10.3389/fphar.2023.1190402

**Published:** 2023-08-04

**Authors:** Ben Boyarko, Sonia Podvin, Barry Greenberg, Jeremiah D. Momper, Yadong Huang, William H. Gerwick, Anne G. Bang, Luisa Quinti, Ana Griciuc, Doo Yeon Kim, Rudolph E. Tanzi, Howard H. Feldman, Vivian Hook

**Affiliations:** ^1^ Skaggs School of Pharmacy and Pharmaceutical Sciences, University of California, San Diego, La Jolla, CA, United States; ^2^ Department of Neurology, Johns Hopkins University School of Medicine, Baltimore, MD, United States; ^3^ Gladstone Institute of Neurological Disease, Gladstone Institutes, San Francisco, CA, United States; ^4^ Departments of Neurology and Pathology, University of California, San Francisco, San Francisco, CA, United States; ^5^ Scripps Institution of Oceanography, University of California, San Diego, La Jolla, CA, United States; ^6^ Conrad Prebys Center for Chemical Genomics, Sanford Burnham Prebys, San Diego, CA, United States; Medical Discovery Institute, La Jolla, CA, United States; ^7^ Genetics and Aging Research Unit, McCance Center for Brain Health, Department of Neurology, MassGeneral Institute for Neurodegenerative Disease, Massachusetts General Hospital, Harvard Medical School, Charlestown, MA, United States; ^8^ Department of Neurosciences and Department of Pharmacology, University of California, San Diego, San Diego, United States; ^9^ Alzheimer’s Disease Cooperative Study, University of California, San Diego, La Jolla, CA, United States

**Keywords:** Alzheimer’s disease, therapeutic, treatment, bumetanide, repurpose, memory, APOE4 and AD risk

## Abstract

Therapeutics discovery and development for Alzheimer’s disease (AD) has been an area of intense research to alleviate memory loss and the underlying pathogenic processes. Recent drug discovery approaches have utilized *in silico* computational strategies for drug candidate selection which has opened the door to repurposing drugs for AD. Computational analysis of gene expression signatures of patients stratified by the APOE4 risk allele of AD led to the discovery of the FDA-approved drug bumetanide as a top candidate agent that reverses APOE4 transcriptomic brain signatures and improves memory deficits in APOE4 animal models of AD. Bumetanide is a loop diuretic which inhibits the kidney Na^+^-K^+^-2Cl^−^ cotransporter isoform, NKCC2, for the treatment of hypertension and edema in cardiovascular, liver, and renal disease. Electronic health record data revealed that patients exposed to bumetanide have lower incidences of AD by 35%–70%. In the brain, bumetanide has been proposed to antagonize the NKCC1 isoform which mediates cellular uptake of chloride ions. Blocking neuronal NKCC1 leads to a decrease in intracellular chloride and thus promotes GABAergic receptor mediated hyperpolarization, which may ameliorate disease conditions associated with GABAergic-mediated depolarization. NKCC1 is expressed in neurons and in all brain cells including glia (oligodendrocytes, microglia, and astrocytes) and the vasculature. In consideration of bumetanide as a repurposed drug for AD, this review evaluates its pharmaceutical properties with respect to its estimated brain levels across doses that can improve neurologic disease deficits of animal models to distinguish between NKCC1 and non-NKCC1 mechanisms. The available data indicate that bumetanide efficacy may occur at brain drug levels that are below those required for inhibition of the NKCC1 transporter which implicates non-NKCC1 brain mechansims for improvement of brain dysfunctions and memory deficits. Alternatively, peripheral bumetanide mechanisms may involve cells outside the central nervous system (e.g., in epithelia and the immune system). Clinical bumetanide doses for improved neurological deficits are reviewed. Regardless of mechanism, the efficacy of bumetanide to improve memory deficits in the APOE4 model of AD and its potential to reduce the incidence of AD provide support for clinical investigation of bumetanide as a repurposed AD therapeutic agent.

## Introduction

Alzheimer’s disease (AD) is a neurodegenerative disease characterized by progressive memory deficits with increasing age-related prevalence. Its genetic, co-morbid pathologies and clinical heterogeneity have posed challenges to successful development of pharmacologic therapies. Currently approved therapeutics include acetylcholinesterase inhibitors for the treatment of symptoms and routine daily functions in patients with mild to severe AD (Birks, 2006; Mehta et al., 2012; Kabir et al., 2019). The N-methyl D-aspartate (NMDA) antagonist memantine improves symptoms in moderate to severe AD (Robinson and Keating, 2006; Kishi et al., 2017).

Tremendous efforts in AD drug discovery and development by scientists in industry and the academic sectors have strived over the past several decades to address this unmet therapeutic need. The amyloid hypothesis has been a dominant focus of therapeutic development with approaches that have targeted reducing its production, preventing aggregation, and enhancing clearance to reduce the neurotoxicity of accumulated Aβ peptides and amyloid deposits. Progress has been slow and there have been numerous unsuccessful development programs. In 2021, the monoclonal antibody (mAb) aducanumab that targets the removal of aggregated more so than soluble species of Aβ was granted accelerated approval by the FDA based on data showing clearing of amyloid as a surrogate endpoint but without certainty of significant efficacy to improve the cognitive deficits and course of AD (Cummings et al., 2021; Budd Haeberlein et al., 2022; Whitehouse et al., 2022). Lecanemab, another mAb that lowers protofibrillar amyloid aggregates (Hayato et al., 2022; Prillaman, 2022), recently received accelerated FDA approval having shown more convincing clinical efficacy while also impacting its surrogate endpoint of reduced amyloid plaques (van Dyck et al., 2023). Donanemab is an IgG1 antibody directed to an N-terminal pyroglutamate Aβ epitope and its evaluation in late stage clinical trials found that it improved cognition and lowered amyloid (Mintun et al., 2021). Prior to these successes, a large number of programs have not reached late stage development despite having achieved the preclinical milestone of lowering Aβ (Gu et al., 2017; Loureiro et al., 2020; Moussa-Pacha et al., 2020; Avgerinos et al., 2021; McDade et al., 2021; van Bokhoven et al., 2021; Cummings et al., 2022). Strategies to reduce pathogenic tau accumulation in brain have similarly attracted significant therapeutic development attention but with a low success rate (Congdon and Sigurdsson, 2018; Cummings et al., 2019).

With the difficulties in achieving single target success for AD, there is an increasing appreciation of the complexity of pathway interactions to impact on pathogenic processes. In turn, this has increased interest in utilizing multi-pronged approaches addressing inflammation, oxidative stress, proteostasis, synaptic plasticity, vasculature, receptors, and metabolic mechanisms of AD (Cummings et al., 2022). These approaches lend themselves particularly well to the consideration of repositioned and repurposed medicines rediscovered by *in silico* computational methods leveraging transcriptomic, genetic, and other human omic databases combined with human 3D organoid model systems in high throughput drug screening (Choi et al., 2014; Choi et al., 2015) and clinical pharmacoepidemiology of drug outcomes in large population data sets (Varma et al., 2022). Furthermore, the application of precision medicine targeting subgroups of the population based on unique profiles and risk factors can be used to improve trial design and drug testing.

The APOE4 gene allele is a major risk factor of late onset AD in non-Hispanic whites, with varying degrees of risk in other population groups. The APOE4 allele (Liu et al., 2013; Safieh et al., 2019; Yamazaki et al., 2019; Serrano-Pozo et al., 2021) has been reported to have a prevalence of 48% in AD populations (Ward et al., 2012) and 25% in the general population (Gharbi-Meliani A et al., 2021). Recently, computational evaluation of APOE genotype-dependent transcriptomics signatures of human AD brain combined with query of a drug database of transcriptomic signatures identified bumetanide as a top-scoring drug that reverses transcriptomic AD signatures of the *APOE4* genotype (Taubes et al., 2021). Notably, bumetanide treatment of APOE4 mice rescued memory deficits. Analysis of electronic health records found that bumetanide exposure is associated with a significant reduction in AD incidence. These findings support further investigation of bumetanide as a candidate drug for AD with APOE4.

This comprehensive review provides information and analysis of the suitability of bumetanide drug properties as a potential AD treatment, including reference to other neurological conditions for which bumetanide has also attracted interest. This evaluation of bumetanide covers its blocking action of Cl^−^ uptake mediated by the Na^+^-K^+^-Cl^−^ cotransporter NKCC1 in various kinds of brain cells. Notably, while numerous studies have proposed a (selective) block of neuronal NKCC1 by bumetanide, this is an effect that cannot be achieved with any available drugs because of the ubiquitous nature of NKCC1 expression in cells within and outside the CNS (Russell, 2000). Bumetanide also acts at the NKCC2 transporter thereby inducing diuresis. The pharmacokinetic properties of bumetanide (poor brain penetration and fast metabolism) (Kharod et al., 2019; Löscher and Kaila, 2022) result in very low and transient concentration levels in the brain, suggesting that some of its modes of action are based on non-NKCC1 mechanisms in neurons and/or other brain cells. Finally, it is possible that some of bumetanide’s therapeutic actions described in the literature are “peripheral,” i.e., mediated by cells outside brain tissue, such as those in epithelia, vasculature and the immune system (Löscher and Kaila, 2022). These compiled data suggest that bumetanide action includes non-NKCC1 brain mechanisms in AD deficits.

## Identification of bumetanide for ameliorating APOE4-related AD deficits by precision medicine and computational strategies

**FIGURE 1:**
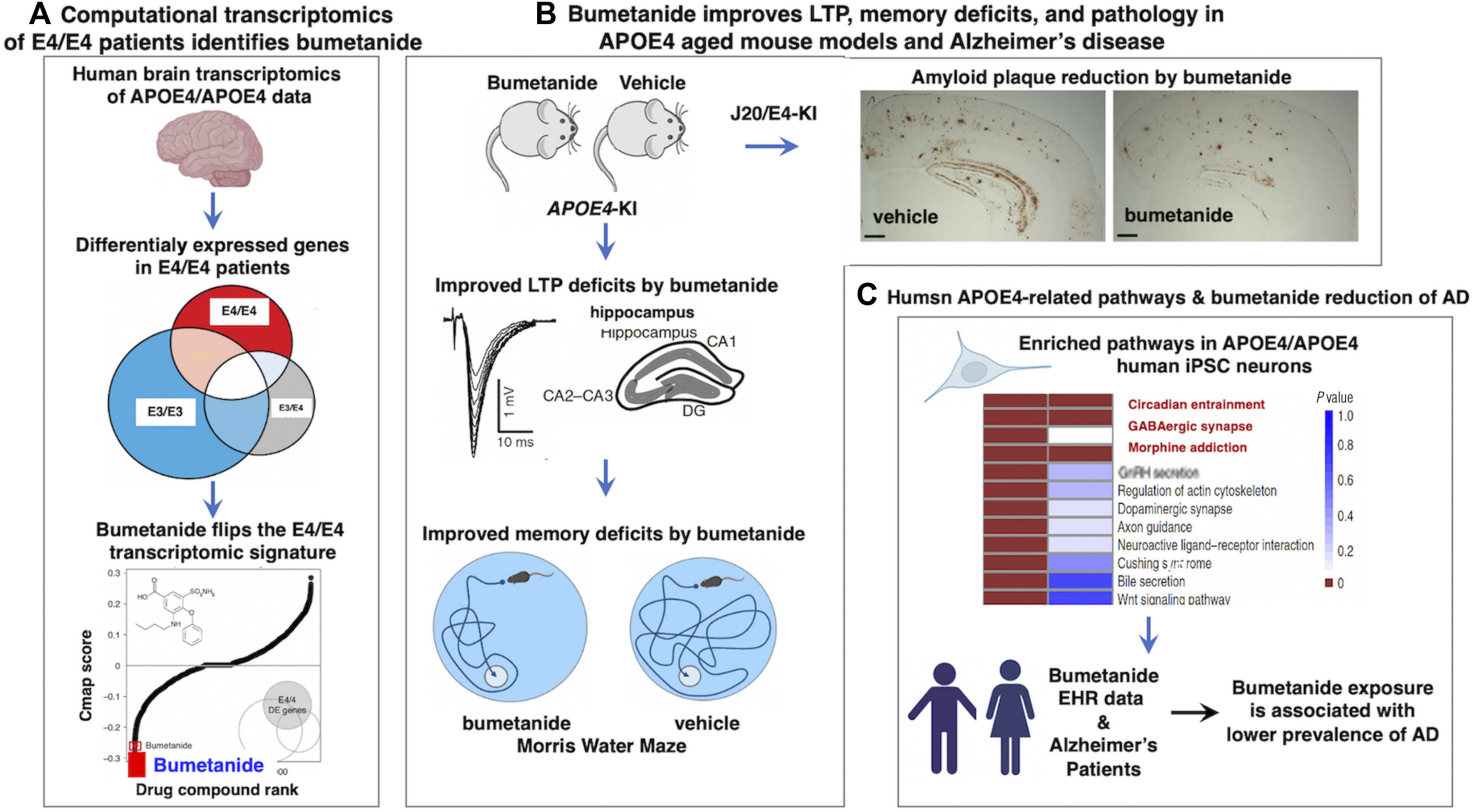
Human computational precision medicine strategy identifies bumetanide for repurposing in APOE4-related Alzheimer’s disease. **(A)** Bumetanide reverses the human APOE4 transcriptomic signatures, achieved by computational precision medicine. Studies of Taubes et al., 2021, assessed human brain temporal lobe tissue transcriptomics data from the GEO resource by stratification by *APOE* genotypes of E4/4, E3/4, and E3/3 to assess the E4 allele risk factor of Alzheimer’s disease (AD). Differential expression signatures were observed in E4/4 AD compared to E3/4 AD and E3/3 AD genotypes, illustrated by gene expression profiles that were downregulated and upregulated that defined dysregulated pathways in AD. Computational analysis of Cmap for drug-induced reversal of E4 transcriptomics signatures identified bumetanide as the top FDA-approved drug for repurposing as a candidate AD therapeutic agent. **(B)** Bumetanide treatment of APOE4-KI mice ameliorates functional and pathological brain deficits. Upon administration of bumetanide to APOE4-KI mice, there was a reduction in LTP deficits of hippocampus and improved memory deficits assessed by the Morris water maze test. In J20/E4-KI AD mice, bumetanide treatment substantially reduced amyloid plaque load in brain. **(C)** Human E4/E4 enrichment of molecular pathways and patients exposed to bumetanide displayed reduced AD prevalence. Human E4/E4 iPSC neurons displayed enrichment in pathways of circadian entrainment, GABAergic synapse, and morphine addiction. Electronic health records (EHR) indicated that patient exposure to bumetanide was associated with lower AD prevalence.

Bumetanide recently emerged as a top repurposed FDA-approved drug candidate for the treatment of APOE4-related AD (Taubes et al., 2021) (Figure 1). Human APOE4 cortical brain transcriptomic signatures (from 97 AD patients and 116 controls) were computationally analyzed with the Connectivity Map (CMap) database to identify drugs that can reverse the APOE4-specific transcriptomic signatures of AD. Cmap contains drug-induced transcriptomic data induced by more than 1,300 drugs. This computational analysis revealed bumetanide as the top scoring drug that reverses human APOE4-specific transcriptomic signatures of AD. Bumetanide treatment of human APOE4 knock-in (KI) mice (homozygous APOE4/APOE4) resulted in improved memory deficits assessed by the Morris water maze assay, and reduced brain electrophysiological deficits in hyperactivity and long-term potentiation (LTP). In J20/APOE4 mice (APP_FAD_, human APP-751/770-Swe-Ind), bumetanide treatment reduced Aβ plaque accumulation and rescued hyperactivity (Taubes et al., 2021). In bumetanide-treated human iPSC neurons, upregulated genes in APOE4/APOE4 neurons were shifted downward by bumetanide. Conversely, those genes that were downregulated were shifted upward by drug treatment. Importantly, in several large EHR databases bumetanide exposure was associated with a 35%–75% lower prevalence of AD in individuals over 65 years of age in cross-sectional single time-point analyses (Taubes et al., 2021). These novel findings support bumetanide as a candidate FDA-approved drug for repurposing as a potential therapeutic agent for AD.

## Bumetanide inhibition of the brain NKCC1 transporter modifies neuronal chloride regulation and GABAergic signaling

**FIGURE 2:**
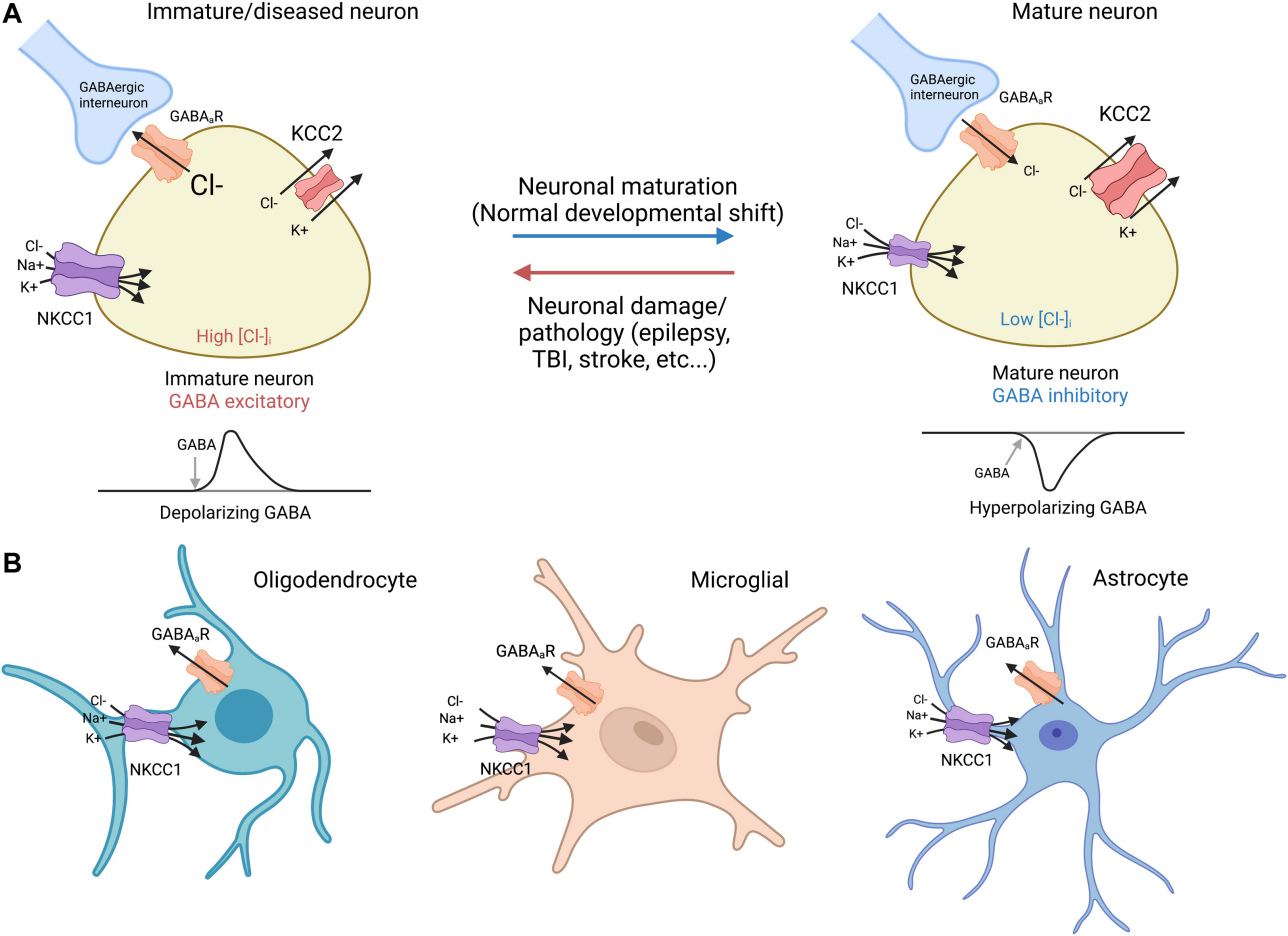
NKCC1 and KCC2 regulate GABAergic transmission in neurons and glia **(A)** Neurons: immature and mature. Immature neurons: In immature neurons, high NKCC1 expression and low levels of KCC2 result in high intracellular Cl^−^, resulting in a net Cl^−^ outflow and subsequent depolarization via activation of GABAergic receptors (Payne et al., 2003). NKCC1 utilizes the Na^+^/K^+^ ATPase to drive Cl^−^ transport against its electrochemical concentration gradient. Mature neurons: During normal neurodevelopment, KCC2 expression increases, leading to low intracellular [Cl^−^], and net inflow of Cl^−^ into the GABA cells by GABAergic receptors, a hyperpolarizing response that decreases firing threshold (Payne et al., 2003). Virtually all mature neurons afflicated by damage or diseases such as epilepsy, TBI, and stroke demonstrate downregulated KCC2 with or without concurrent NKCC1 upregulation similar to expression ratio profiles found in immature neurons (Rivera et al., 2002; Huberfeld et al., 2007). It is noted that NKCC1 exists as splice isoforms of NKCC1a and NKCC1b that are primarily non-neuronal and neuronal, respectively (Kurki et al., 2022). **(B)** Glia cells: oligodendrocyte, microglia, and astrocyte. NKCC1 is expressed in oligodendrocytes (Wang et al., 2003; Yu et al., 2018), microglia (Luo et al., 2021; Tóth et al., 2022), and astrocytes (Su et al., 2002). KCC2 expression is absent in glia cells (Huang et al., 2023). GABAergic receptor expression has been observed in all glia cells, including oligodendrocytes (Williamson et al., 1998; Arellano et al., 2016), microglia (Favuzzi et al., 2021; Logiacco et al., 2021), and astrocytes (Yoon et al., 2012; Mederos et al., 2021).

In developing and diseased CNS neurons, NKCC1 regulates intracellular Cl^−^ levels together with the KCC2 K^+^-Cl^−^ cotransporter (Figure 2). In immature neurons, the functionally excitatory GABA_A_ receptor mediated transmission (Ben-Ari, 2002) is attributable to NKCC1-mediated Cl^−^ uptake, while upregulation of the neuron-specific K^+^-Cl^−^ cotransporter KCC2 (which transports Cl^−^ out of the cell) promotes hyperpolarizing GABA responses (Kaila et al., 2014). Notably, both KCC2 and NKCC1 are expressed as two splice isoforms. With regard to KCC2, out of the two splice variants KCC2a and KCC2b (Kaila et a 2014), the latter is responsible for the hyperpolarizing developmental shift (Rivera et al., 1999). Recent work (Kurki et al., 2022) has shown that the developmental expression patterns of the two splice variants of NKCC1 (NKCC1a and NKCC1b) are different in neurons and non-neuronal cells in the brain; the non-neuronal splice variant NKCC1a shows an increase during development, while the neuronal splice isoform NKCC1b remains at a constant level and therefore has a negligible effect on GABA signaling in healthy mature neurons (see Virtanen et al., 2020 for discussion).

In numerous pathophysiological conditions such as epilepsy, TBI, and stroke, the dedifferentiation of neurons is associated with a marked increase in NKCC1 expression accompanied by downregulation of KCC2 thus recapitulating the expression profiles found in immature neurons (Payne et al., 2003; Jaenisch et al., 2010; Kharod et al., 2019). With respect to AD, Aβ42 increases NKCC1 in mouse hippocampus (Lam et al., 2022), and APP expression decreases KCC2 in cortical cells without modifying NKCC1 (Doshina et al., 2017). Such research suggests that elevated NKCC1 with diminished KCC2 may predict an altered balance of excitatory to inhibitory (E/I) activity towards excitatory GABAergic signaling. Further examination of the changes in NKCC1 and KCC2 activities in mature and immature neurons will provide new knowledge of chloride ion regulation.

As stated already above, NKCC1 is expressed in all brain cells, including all glia cell types (Kharod et al., 2019; Löscher and Kaila, 2022) such as oligodendrocytes (Wang et al., 2003; Yu et al., 2018), microglia (Luo et al., 2021; Tóth et al., 2022), and astrocytes (Su et al., 2002). Notably, there is extensive evidence demonstrating GABAergic receptor expression in oligodendrocytes (Williamson et al., 1998; Arellano et al., 2016), microglia (Favuzzi et al., 2021; Logiacco et al., 2021), and astrocytes (Yoon et al., 2012; Mederos et al., 2021).

In both APOE4-KI and J20/APOE4-KI models of AD in mice, hippocampal brain neurons display hyperexcitability assessed in electrophysiological studies (Taubes et al., 2021). Treatment with bumetanide for 12 weeks (ip, 0.2 mg/kg, once per day) rescued the pathophysiological hyperexcitability and the deficits in long-term potentiation (LTP) in both APOE4-KI and J20/APOE4-KI mice. The rescue of LTP by bumetanide was accompanied by amelioration of cognitive deficits in APOE4-KI mice as measured by the Morris water maze test (Taubes et al., 2021).

## Bumetanide effects mediated by NKCC1 in glia and endothelial cells

Glia cells of the oligodendrocyte type express high levels of NKCC1 (Kurki et al., 2022). Bumetanide significantly reduced AMPA-mediated excitotoxicity in cultured oligodendrocytes, including Na^+^ influx and oligodendrocyte swelling (Chen et al., 2007). Interestingly, bumetanide treatments (0.3 mg/kg, ip once daily) reduced myelin damage and enhanced the proliferation of oligodendrocytes in mice under ischemic conditions (Yu et al., 2018). Impaired myelination and oligodendrocyte deficits have been documented in AD and APOE4 oligodendrocytes (Blanchard et al., 2022; Hirschfeld et al., 2022). Therefore, it is possible that bumetanide treatment may contribute to ameliorating oligodendrocyte deficits in J20/E4-KI AD mice (Taubes et al., 2021). Indeed, bumetanide treatments (0.2 mg/kg, once daily ip injection for 12 weeks) significantly reversed transcriptomic perturbation signatures in J20/E4-KI oligodendrocytes (cluster 9) and oligodendrocyte progenitor cells (cluster 6), as well as neuron and astrocyte clusters (Taubes et al., 2021).

Microglia are the resident innate immune cells in the brain (Lenz and Nelson, 2018) and play a critical role in the pathogenesis of AD (Zhang et al., 2021). NKCC1 expression has been observed in microglia of embryonic and adult brains (Kurki et al., 2022), and the genetic ablation of microglia NKCC1 in mice accelerates transformation of microglia to reactive state. Bumetanide applied systemically (2 mg/kg, ip) decreases inflammatory responses (G-CSF, KC, IL-1β, and IL-1α) in the mouse brain upon intracortical LPS administration (Tóth et al., 2022). Interestingly, direct intracortical brain injection of bumetanide (50 μM with 200 μL final volume), together with LPS, markedly potentiates LPS-induced G-CSF, KC, IL-1β, and IL-1α increases in the brain, displaying a clear contrast with ip injection. These data show two different tracks of bumetanide regulation of brain microglia functions through direct administration of bumetanide into the brain as well as a peripheral therapeutic mode of action of bumetanide which might be relayed to the brain via immune signalling.

Bumetanide treatment (5–10 µM) significantly reduced Na^+^, Ca^2+^ and Cl^−^ influx in astrocytes following *in vitro* ischemia by blocking NKCC1 activity (Lenart et al., 2004). The uncontrolled Na^+^/Cl^−^ influx can result in water influx and astrocyte swelling, which could damage astrocytes and lead to brain edema (Jayakumar et al., 2008).

NKCC1 is also located in the luminal membrane of blood-brain barrier (BBB) endothelial cells Foroutan et al., 2005), regulating vasogenic edema formation and BBB disruption during brain ischemia and traumatic brain injury (TBI) (Foroutan et al., 2005; Simard et al., 2010; Zhang et al., 2017). Bumetanide treatments (iv and ip) significantly decreased the contusion volume and brain edema (Lu et al., 2006; Simard et al., 2010).

## Distribution of NKCC1 and KCC2 in human brain

**FIGURE 3:**
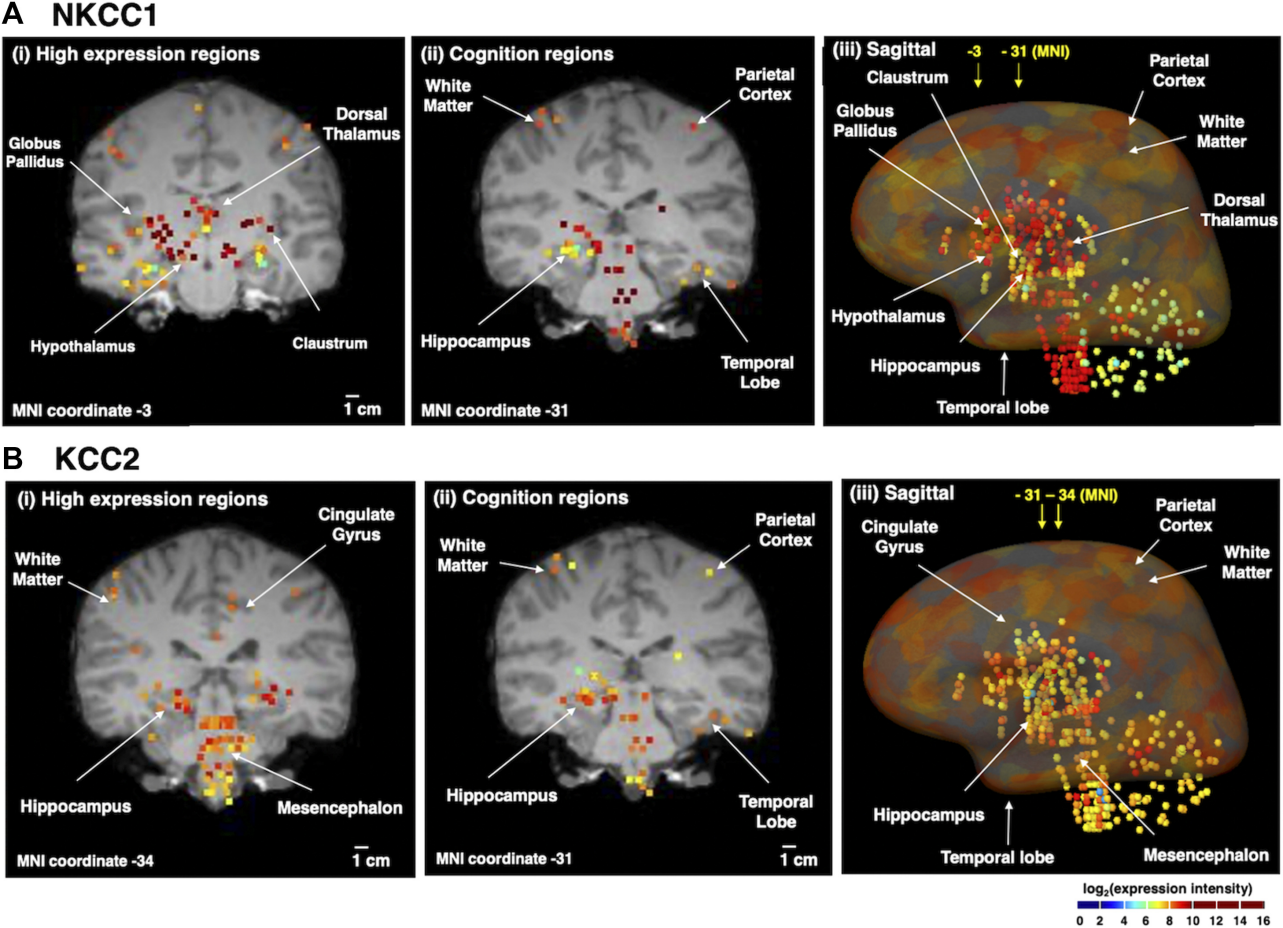
Human brain distribution of NKCC1 and KCC2 expression. NKCC1 and KCC2 microarray gene expression data of 169 distinct brain regions were acquired from the Allen Human Brain Map resource (human.brain-map.org) (Sunkin et al., 2013). The log_2_ expression intensities were overlaid on coronal MRI images of an adult brain. Quantitative expression values ranged from log_2_ (intensity) values of 2–10, illustrated by the colored heatmap ranging from low expression show in blue, to high expression shown in red. The yellow to red colored areas show regions with high expression levels. It is noted that the apparent colocalization at the present resolution does not indicate cellular colocalization of the two transporters (for further discussion, see main text). **(A)** NKCC1 expression in human brain regions. The distribution of NKCC1 gene expression in human brain regions is illustrated for (i) high levels of expression (coronal view), (ii) expression in regions involved in cognition (coronal view), and (iii) sagittal view of the distribution of expression. **(B)** KCC2 expression in human brain regions. The distribution of KCC2 gene expression in human brain regions is illustrated for (i) high levels of expression (coronal view), (ii) expression in regions involved in cognition (coronal view), and (iii) sagittal view of the distribution of expression.

The distribution of NKCC1 mRNA in adult human brain was compared to KCC2 mRNA (Figure 3). Quantitative microarray gene expression data among 169 human brain regions were acquired from the Allen Institute Human Brain Map resource (human.brain-map.org) that provides expression data for all human genes. Gene expression levels (expressed as log_2_ intensity) are viewed on coronal MRI brain images (Figure 3).

Immunohistochemical work has shown that KCC2 is expressed in practically all neurons in the brain, with only a few exceptions such as the substantia nigra in the striatum (Gulácsi et al., 2003) and the reticular nucleus of the thalamus (Barthó et al., 2004). Also, NKCC1 is expressed (as emphasized above) in all brain cell types. Thus, the human gene expression data illustrated in Figure 3 highlights regions with apparently high expression levels of these two transporters. Moreover, the gene expression data does not assess possible cellular colocalization of the two transporters or the cell types of expression.

With the above caveats in mind, it is still of interest to note that NKCC1 displays high expression in numerous deeper brain regions (Figure 3A) including the globus pallidus, dorsomedial nucleus of thalamus, hypothalamus, and claustrum. All brain regions contain heterogeneous neuronal and glia cell types that may express NKCC1. In the dorsomedial nucleus that contains NKCC1, information is relayed between the limbic system and the prefrontal cortex for roles in attention, planning, organization, and active memory. In brain regions involved in cognition, NKCC1 expression occurs in parietal cortex, temporal lobe, and hippcampus. A sagittal view of expression reinforces coronal section data showing high NKCC1 expression in cortical areas, globus pallidus, hypothalamus, hippocampus, dorsal thalamus, temporal lobe, brainstem, and also white matter.

Examination of KCC2 expression in human brain regions (Figure 3B) shows that high levels are expressed in hippocampus, mesencephalon, and cingulate gyrus. KCC2 expression is known to be present in only neurons, rather than glia cells (Williams et al., 1999; Huang et al., 2023). KCC2 is present in regions that regulate cognitive functions consisting of cortical areas and hippocampus, and is also abundantly expressed in mesencephalon and brainstem. These findings are complementary to studies of KCC2 protein expression with immunohistochemistry data (Sedmak et al., 2016).

Given that ion regulation provides the major energetic load on brain tissue (Buzsáki et al., 2007), the regions with high overlap of NKCC1 and KCC2 mRNA expression in Figure 3 might reflect at least to some extent a locally high energetic demand. It will be of interest to compare NKCC1 and KCC2 expression levels in distinct brain regions *in vivo* (e.g., Figure 3) as well as in neuronal and glia cells in post-mortem human AD brain during progression of the disease.

## Estimated bumetanide brain levels that improve memory deficits in APOE4 mice suggest non-NKCC1 mechanisms

### Bumetanide efficacy to improve memory deficits occurs at brain drug concentrations below levels that inhibit NKCC1

To address the potential of bumetanide to ameliorate impairment of memory deficits in AD, it is important to determine whether the bumetanide concentrations in brain achieve IC_50_ levels that inhibit the NKCC1 transporter, or whether its concentrations are below this level. Bumetanide efficacy occurring at brain levels below NKCC1 IC_50_ values implicates non-NKCC1 mechanisms for possible utility of this drug in AD.

**FIGURE 4:**
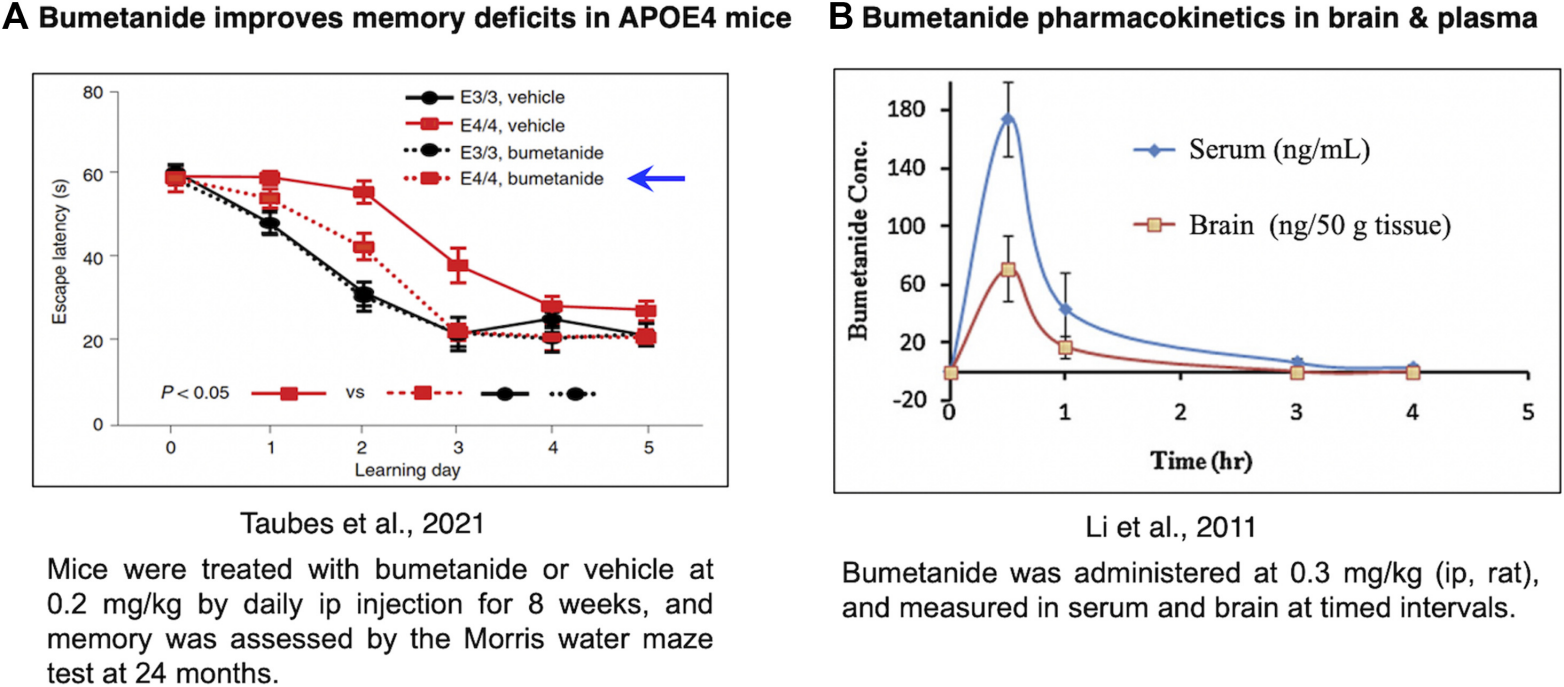
Bumetanide ameliorates memory deficits in APOE4-KI mice at drug doses that provide measurable levels of bumetanide in brain. **(A)** Efficacy of bumetanide for ameliorating memory deficits. Mice were treated with bumetanide or vehicle at 0.2 mg/kg by daily ip injection for 8 weeks, and memory was assessed by the Morris water maze test at 24 months. Latency times are shown as mean ± s.e.m (*n* = 11 animals per group) (Taubes et al., 2021). **(B)** Pharmacokinetics of bumetanide in brain and serum. Bumetanide was administered at 0.3 mg/kg (ip, rat), and measured in brain and serum at timed intervals of 0.5, 1, 3 and 4 h (Li et al., 2011).

Bumetanide was found to improve memory deficits in the APOE4 mouse model after drug administration of 0.2 mg/kg by intraperitoneal (ip) injection once a day for 8 weeks (Figure 4A) (Taubes et al., 2021). At a similar dose of 0.3 mg/kg, bumetanide was present in the brain with peak levels observed at 30 min after ip administration (Li et al., 2011) (Figure 4B). At the dose range of 0.15–0.5 mg/kg (ip) in rats, brain levels of bumetanide were 1–27 nM, below the IC_50_ levels of 100–300 nM bumetanide needed to significantly inhibit NKCC1 (Table 1) (Puskarjov et al., 2014; Löscher and Kaila, 2022). At higher doses in the range of 2–10 mg/kg delivered intravenously (iv) in mice and rats, bumetanide levels in the brain were measured as 160–1,040 nM, which falls within the IC_50_ range of 100–300 nM bumetanide required to inhibit NKCC1 (Table 1). These data show that at 0.2 mg/kg bumetanide, improvement of memory deficits in the APOE4 mice (Taubes et al., 2021) would be estimated to occur at brain levels insufficient to inhibit NKCC1. Significantly, these pharmacokinetic data suggest that at 0.2 mg/kg, bumetanide improvement of memory deficits in the APOE4 mice (Taubes et al., 2021) likely results from non-NKCC1 mechanisms.

**TABLE 1:** Bumetanide brain levels measured after peripheral doses administered to rodents.

Compound	Dose	Species	Age/sex	Health status	Time of sacrifice after drug admin	Average brain levels	% Plasma drug reaching brain (brain to plasma ratio x100)		NKCC1 inhibitory levels of 100–300 nM^a^ of bumetanide reached?	References
Bumetanide	0.15 mg/kg i.p	Rat	P10/male	Normal	0.5 h	0.51 ng/g (1.4 nM) [at 10 min 0.76 ng/g (2.11 nM)]		0.43%	No	Cleary et al. (2013)
Bumetanide	0.15 mg/kg i.p	Rat	P10/male	15 min after hypoxia/neonatal seizures	0.5 h	0.68 ng/g (1.8 nM)		0.5%	No	Cleary et al. (2013)
Bumetanide	0.3 mg/kg i.p	Rat	P10/male	Normal	0.5 h	0.94 ng/g (2.6 nM) [at 10 min 1.2 ng/g (3.4 nM)]		0.55%	No	Cleary et al. (2013)
Bumetanide	0.3 mg/kg i.p	Rat	P10/male	15 min after hypoxia/neonatal seizure	0.5 h	1.07 ng/g (27 nM)		0.5	No	Cleary et al. (2013)
Bumetanide	0.3 mg/kg i.p	Rat	P11/both sexes	∼10 min after asphyxia/neonatal seizures	∼1 h	10 ng/g (27 nM)		3.6%	No	Johne et al. (2021b)
Bumetanide	0.3 mg/kg i.p	Rat	Adult/male	Normal	0.5 h	∼1.4 ng/g (3.8 nM)		0.68%	No	Li et al. (2011)
Bumetanide	0.5 mg/kg i.p daily for 3 weeks	Rat	P10/both sexes	Normal	0.5 h after last drug injection	1.7 ng/g (4.7 nM)		0.46%	No	Wang et al. (2015)
Bumetanide	0.5 mg/kg i.p daily for 3 weeks	Rat	P10/both sexes	Hypoxia/neonateseizures	0.5 h after last drug injection	2.2 ng/g (6 nM)		0.77%	No	Wang et al. (2015)
Bumetanide	2 mg/kg i.v	Mouse	Adult	Normal	5 min (C_max_)	∼60 ng/g (160 nM)		∼0.5%	Yes	Savardi et al. (2020)
Bumetanide	10 mg/kg i.v	Rat	Adult/female	Normal	0.25 h (C_max_)	53 ng/g (150 nM) (cortex and amygdala)		1.4%	Yes	Töllner et al. (2015)
Bumetanide	10 mg/kg i.v	Rat	Adult/female	Normal	0.25 h (C_max_)	11.8 ng/g (320 nM) (cortex and amygdala)		1.4%	Yes	Tôpfer et al. (2014)
Bumetanide	10 mg/kg i.v, then i.v. with 6 mg/kg/h over 5 h	Rat	Adult/male	Normal	Hippocampal dialysate, drug measured after 5-h drug infusion	1,370 ng/g (3,760 nM) hippocampus; 132 ng/mL (360 nM) in dialysate		∼6% for whole tissue but only ∼0.01% for dialysate (extracellular) levels	Yes (also in the extracellular fluid)	Donovan et al. (2015)
Bumetanide	10 mg/kg i.v	Mouse	Adult/female	Normal	0.5 h	330 ng/g (910 nM)		1.6%	Yes	Töllner et al. (2015)
Bumetanide	10 mg/kg i.v	Mouse	Adult/female	Normal	0.5 h	270 ng/g (740 nM)		1.8%	Yes	Töllner et al. (2015)
Bumetanide	10 mg/kg i.v	Mouse	Adult/female	Normal	0.25 h	380 ng/g (1,040 nM)		1.1%	Yes	Römermman et al. (2017)
Bumetanide	10 mg/kg i.v	Mouse	Adult/female	Normal	2 min (C_max_)	1,030 ng/g (2,830 nM)		1.5%	Yes	Hampel et al. (2021)
Bumetanide	10 mg/kg i.v	Rat	P11/both sexes	∼10 min after asphyxia/neonatal seizures	∼1 h	190 ng/g (520 nM)		2.5%	Yes	Johne et al. (2021a)

^a^ Bumetanide at 100–300 nM has been reported to represent the minimal concentrations to inhibit NKCC1 (Löscher and Kaila, 2022). It is noted that the reported IC_50_ values of bumetanide have variability from study to study (Hampel et al., 2018; Löscher and Kaila, 2022).

It will be useful for future studies to characterize the free bumetanide brain levels compared to bumetanide that may be bound to proteins and lipids (Löscher & Kaila, 2022). Such data would suggest even lower concentrations of bumetanide in brain that are consistent with non-NKCC1 mechanisms of bumetanide to ameliorate memory deficits.

### Mechanisms of bumetanide action involving pathways that regulate GABAergic signaling, circadian entrainment, and morphine addiction

Three distinct pathways have been suggested based on RNA-seq data of bumetanide-treated APOE4-KI mice and APOE4 iPSC-derived neurons (Taubes et al., 2021) consisting of GABAergic transmission, circadian entrainment, and morphine addiction pathways. While bumetanide inhibits NKCC1 at 100–300 nM, regulation of the GABAergic system at nM levels could occur through non-NKCC1 mechanisms involving features of the complex regulatory networks of neurotransmission.

Circadian entrainment and morphine addiction represent two pathways of the limbic system, a group of cortical and subcortical structures involved in memory, emotion, reward, and motivation. Circadian systems regulate sleep which is disturbed in AD patients (Musiek and Holtzman, 2016). Impairment of limbic system networks contribute to the pathological heterogeneity of AD. For example, regions such as the suprachiasmatic nucleus of the hypothalamus (responsible for sleep and circadian rhythms) have displayed neuroinflammation induced by dysregulated GABA levels (Roy et al., 2019; Singer and Alia, 2022). Similarly, disruptions in regions such as the ventral tegmental area (responsible for reward pathways) and memory impairments have been shown in patients with early AD (Bozzali et al., 2016; Bozzali et al., 2019) and degeneration of dopaminergic neurons in the VTA also precede the deposition of Aβ plaques in the hippocampus (Alves et al., 2017).

Importantly, bumetanide treatment rescues transcriptomic perturbations in all three pathways of GABAergic signaling, circadian entrainment, and morphine addiction pathways in hippocampal neurons and glia cells of aged APOE4-KI and APOE4 iPSC-derived neurons (Taubes et al., 2021). The collection of these data suggest that bumetanide likely targets non-NKCC1 effectors related to neuroinflammation and memory impairments that contribute to AD pathologenesis. Further investigation is warranted to understand how bumetanide alters these and other downstream pathways that contribute to disruptions of normal CNS homeostasis in AD.

## Bumetanide doses and brain levels associated with improved functions in animal models of neurological diseases

Bumetanide has attracted broad interest in models of neurological disorders including ischemic stroke, autism with Rett Syndrome and Fragile X, as well as traumatic brain injury. These data can shed additional light on the relevant CNS mechanisms of action of bumetanide through inhibition of NKCC1 and/or non-NKCC1 mechanisms in various disease contexts. These analyses provide an understanding of the range of efficacious bumetanide brain levels that improve behavioral outcomes of brain disorders, including memory impairment that occurs in ischemic stroke related to AD.

### Ischemic stroke improvements by bumetanide, including rescue of memory impairment

In models of ischemic stroke, bumetanide at the doses of 0.3 and 0.5 mg/kg by daily ip for 3–4 weeks resulted in improved memory and brain outcomes (Wang et al., 2015; Yu et al., 2018) (Table 2). This dose range is similar to that used for improvement of memory deficits in the APOE4 AD model that used 0.2 mg/kg bumetanide (Taubes et al., 2021).

**TABLE 2:** Bumetanide (BTN) dosing and efficacious outcomes in neurological deficits in animal models.

Neurological disease model	Experimental paradigm	Bumetanide dose and timing	Number of BTN doses	Dose delivery	Strain	Age and sex	Outcome	References
Alzheimer’s Disease	APOE4-KI and J20/APOE4 mouse models of AD	0.2 mg/kg	Once daily for 8 or 12 weeks	*In vivo*, i.p	Mice, APOE4-KI, J20/APOE4	22 months APOE4-KI, 10 months J20/APOE4 mice, female	Bumetanide treatment of APOE4-KI mice rescued memory loss and improved LTP. Treatment of J20/APOE4 mice reduced Aβ plaque load and rescued AD-like neuronal excitability	Taubes et al. (2021)
Ischemic stroke	Bilateral common carotid artery stenosis (BCAS)	0.3 mg/kg	Once daily, 28 days before BCAS	*In vivo*, i.p	Mice	9–11 weeks, male	Amelioration of white matter lesions and cognitive impairment	Yu et al. (2018)
Hypoxia, Ischemia	Graded global hypoxia for 15 min in an airtight chamber	0.5 mg/kg	Once a day for 3 weeks	*In vivo*, i.p	Wistar rats	P10 (on day of hypoxia), unspecified sex	Reduction in EEG seizures. Increased neurogenesis of DGC cells after hypoxia-induced seizures	Wang et al. (2015)
Ischemic stroke	Permanent left middle cerebral artery (MCAO) occlusion	7.6–30.4 mg/kg 20 min before MCAO	1–4 doses	*In vivo*, i.v	Sprague-Dawley rats	unspecified	Bumetanide reduced edema	O’Donnell et al. (2004)
Rett Syndrome, autism	*Mecp2* ^*-/y*^ hemizygous KO mice	0.2 mg/kg	Once daily for 3–4 days, starting at P10	*In vivo*, i.p	*Mecp2* ^*-/y*^ hemizygous KO mice (species unspecified)	P10-P14	Disequilibrium E/I potential for GABA is restored by bumetanide in Mecp2 gene hemizygous KO mice	Banerjee et al. (2016)
Fragile X (FRX),autism	Exposure *in utero* to bumetanide in mice carrying the fragile X mutation (FRX)	2–2.5 mg/kg pretreatment of maternal animals	1	*In vivo, in drinking water*	Wistar rats, mouse strain not specified	E18, P0, P2, P4, P7, P8, P15, and P30 (mice); E20, P0, P2, P4, P7, P15, and P30 (rats)	Bumetanide treatment of FRX pregnant mothers resulted in (1) normal oxytocin-mediated reduction of Cl-, (2) switch of GABA from excitatory to inhibitory in FRX offspring, and (3) restoration of aberrant autistic behavior	Tyzio et al. (2014)
Traumatic brain injury	hemilaminectomy at lumbar L4-L6	30 mg/kg, 1 h before surgery	1	*In vivo, i.p*	Wister rats	Adult male	Bumetanide prevents TBI neuronal death	Shulga et al. (2012)
Traumatic brain injury	Single, closed-head, unilateral cortical injury	30 mg/kg, 3 days after TBI	1	*In vivo, ip*	Wildtype C57BL/6 mice	6–8 weeks, unspecified sex	Suppression of TBI-induced seizures	Wang et al. (2017)
Traumatic brain injury	CCI	2 mg/kg, 30 min before CCI.	1	*In vivo, i.p*	Wildtype C57BL6/J mice	Adult male, 2 months	Reductions in neuronal swelling and increased n network excitability	Sawant-Pokam et al. (2020)
Traumatic brain injury	Controlled cortical impact (CCI)	15 mg/kg, 20 min before CCI	1	*In vivo, iv*	Wildtype C57BL/6 mice	Embryonic E14-15 and adult of 12 weeks unspecified sex	Bumetanide attenuated TBI brain edema and neurological behavioral score	Hui et al. (2016)
Traumatic brain injury	Weight drop (450 g, 2 m height)	15 mg/kg	1	*In vivo, iv*	Wistar rat	Adult, male	Bumetanide attenuates TBI-induced neuronal loss and edema	Lu et al. (2007)
Intracerebral hemorrhage (ICH)	Collagenase model (collagenase infusions into left striatum)	40 mg/kg	1 dose every 6 or 12 h	*In vivo, oral, i.p*	Sprague-Dawley rats	2–4 months, unspecified	Minor reduction in edema after early dosing, but no improvement in behavior or lesion volume	Wilkinson et al. (2019)

In a study of ischemic stroke modeled by bilateral common carotid artery stenosis (BCAS) (Yu et al., 2018), bumetanide administered at 0.3 mg/kg, ip once daily to mice for 28 days resulted in amelioration of brain white matter lesions and improved memory. This bumetanide dose of 0.3 mg/kg is predicted to result in brain levels of 2–30 nM based on measurements in several studies of rodents receiving 0.3 mg/kg bumetanide (Table 1). Clearly 2–30 nM is below the 100–300 nM levels of bumetanide needed for inhibition of NKCC1.

In a neonate hypoxia model (rat), bumetanide at 0.5 mg/kg, ip daily for 3 weeks, resulted in reduced seizures and promoted neurogenesis of dentate gyrus cells (DGCs) (Wang et al., 2015) (Table 2). The peak levels of bumetanide in brain after hypoxia were 2.17 ng/mL, corresponding to 6 nM bumetanide which was higher than non-hypoxic controls of 4.6 nM bumetanide (30 min after ip). Again., these brain levels of bumetanide are much lower than 100–300 nM needed for inhibition of NKCC1 (Russell, 2000; Löscher and Kaila, 2022) (Table 1).

In contrast, a study using substantially higher doses of bumetanide at 7.6–30 mg/kg (iv) given 20 min before MCAO (middle cerebral artery occlusion), a model of ischemia in rats, resulted in decreased MCAO-induced brain edema (O'Donnell et al., 2004) (Table 2). Such high bumetanide doses have been found to result in brain drug levels that are higher (estimated as above 1,000 nM bumetanide) than that needed to inhibit NKCC1 (Table 1).

These studies demonstrated effective improvement of ischemic stroke-induced memory deficits by bumetanide at a low dose (0.3–0.5 mg/kg, once daily ip) likely involving non-NKCC1 mechanisms in models of carotid artery stenosis (Yu et al., 2018) and neonate hypoxia (Wang et al., 2015), but higher doses of 7–40 mg/kg were needed for efficacy in the MCAO ischemia model (O'Donnell et al., 2004) (Table 2). These findings suggest that different ischemia subtypes may involve different combinations of non-NKCC1 and NKCC1 mechanisms.

### Autism of Rett syndrome and Fragile X syndrome

Bumetanide reversed the autistic behavior of isolation-induced ultrasonic vocalizations in newborn mice. In the Rett syndrome (RTT) mouse model of autism expressing loss-of-function mutations in the *Mecp2* gene (methyl-CpG binding protein 2), mice show an increased neuronal excitation-to-inhibition (E/I) ratio (Banerjee et al., 2016) (Table 2). This altered E/I ratio is restored with bumetanide treatment (0.2 mg/kg, ip daily for 3–4 days starting at P10). The 0.2 mg/kg dose of bumetanide is predicted to result in brain levels of 1–2 nM bumetanide, based on reported studies (Table 1).

In mice expressing the FRX mutation, bumetanide treatment at 2–2.5 mg/kg (via drinking water) in pregnant mothers (rats) resulted in improvement of autistic behavior and reversal of FRX-induced hyperexcitability (Tyzio et al., 2014) (Table 2). Bumetanide treatment switched the action of GABA from excitatory to inhibitory in FRX offspring. The bumetanide dose of 2–2.5 mg/kg administered to the mother would be predicted to result in brain levels of 160 nM bumetanide (Table 1) and likely lower levels in the pup brains. Since bumetanide levels in the pup brain are likely to be much lower, predictions of NKCC1 or non-NKCC1 mechanisms will need studies to assess brain drug levels in the pup.

These studies suggest that bumetanide may improve autism behaviors through non-NKCC1 mechanisms in brain in the RTT model (Banerjee et al., 2016) (Table 2). But in the FRX model, a dose of 2–2.5 mg/kg resulting in estimated brain levels of 160 nM may impact both NKCC1 and non-NKCC1 mechanisms (Table 1).

### Traumatic brain injury improvements by bumetanide

In several animal models of traumatic brain injury, administration of bumetanide at 30 mg/kg (ip, given before TBI) improved neuronal loss and reduced edema in brains of rodents subjected to *in vivo* axotomy (hemilaminectomy) (Shulga et al., 2012). This same treatment *w*th bumetanide decreased TBI-induced seizures in a single closed-head TBI model in mice (Wang et al., 2017) (Table 2). The dose of 30 mg/kg (ip bumetanide) would be expected to result in brain levels of the drug above 100–300 nM for effective inhibition of NKCC1 transporter in brain, based on drug levels in brain after ip doses (Table 1) (Löscher and Kaila, 2022). In another study of TBI modeled by controlled cortical impact (CCI) in mice, bumetanide at 2 mg/kg (ip) given 30 min before CCI resulted in decreased neuronal swelling and increased neuronal excitability (Sawant-Pokam et al., 2020) (Table 2).

In two other TBI studies using 15 mg/kg bumetanide (iv) before TBI, the drug resulted in attenuation of neuronal apoptosis and improved the neurological score for behavioral functions (Hui et al., 2016), and resulted in attenuation of neuronal loss and brain edema (Lu et al., 2007) (Table 2). These studies using bumetanide at 15 mg/kg (iv) are expected to result in brain drug levels above that needed to antagonize the NKCC1 transporter (100–300 nM bumetanide) since a similar dose of 10 mg/kg bumetanide results in brain drug levels of 150–1,000 nM (at 15–60 min after iv administration) (Table 1).

These estimates of bumetanide brain levels in such TBI models is based on predictions of the drug administered to non-injury conditions in rodents (Table 1). It is known that TBI results in disruption of the blood-brain barrier (BBB) (Cash and Theus, 2020; Sulhan et al., 2020) that may result in higher drug levels in brain compared to non-injury conditions. It will be useful to assess bumetanide brain levels after TBI conditions.

Overall, these studies of TBI models utilized bumetanide doses that can inhibit NKCC1 in brain and/or the vasculature, and provide support for the beneficial outcomes of bumetanide involving the NKCC1 transporter. Further understanding of bumetanide efficacy and pharmacological properties will be gained from dose-response studies to assess the range of effective doses for TBI.

## Clinical bumetanide doses for efficacious improvement of neurological deficits

Beyond preclinical models, the efficacy of bumetanide in treating patients afflicted with neurological impairments associated with a spectrum of brain disorders has been evaluated. Several of these clinical studies have assessed a range of doses of bumetanide with improved neurological outcomes in autism, schizophrenia, parkinson’s disease, and epilepsy (Table 3).

**TABLE 3:** Bumetanide (BTN) doses and PK parameters of bumetanide to achieve efficacy in human clinical trials.

Neurological disease	Study design	Age and sex	BTN dose	Number of BTN doses	Dose delivery	Study paradigm	Outcomes	Adverse effects	References
Autism	Pilot study (n = 5)	Children ages 3–11 years old	1 mg	Long-term administration	Oral	1.0 mg once daily for 3 months	Bumetanide significantly decreased autistic behavior assessed by CARS, CGI.	No side effects reported	Lemonnier and Ben Ari (2010)
Autism and Asperger syndrome	Placebo controlled, double blind, human (*n* = 60)	Children ages 3–11 years old, M/F	1 mg/day	Long-term administration	Oral	0.5 mg twice a day for 3 months, followed by 1 month washout	Bumetanide reduced autism assessed by CARS and Clinical Global Impression	Side effects of mild hypokalemia treated with supplemental potassium	Lemonnier et al. (2012)
Autism	Phase II dosing-range, placebo controlled double blind (*n* = 88)	Children and Adolescent ages 2–18, M/F	1.0, 2.0 and 4.0 mg	Long-term administration	Oral	0.5, 1.0, and 2.0 twice daily for 3 months	Improvements in CARS, CGI, and SRS scores. Improvement in core symptoms and favorable benefit/risk ratio	Hypokalemia, urine elimination, loss of appetite, dehydration, and asthenia	Lemonnier et al. (2017)
Autism	Single treatment (*n* = 28) and combined treatment (bumetanide and behavior treatment, *n* = 32)	2.5–6.5, M/F	1 mg	Long-term administration	Oral	0.5 mg twice daily for 3 months; combined treatment with ABA training	Total scores of the ABC, CARS, and SI were decreased in both groups after 3 months (*p* < 0.05)	No adverse effects of bumetanide were observed	Du et al. (2015)
Autism	Open-label, pilot study (*n* = 9)	14.8–28.5 years, M/F	1 mg	Long-term administration	Oral	1 mg once daily for 10 months	Bumetanide reduced eye activation of amygdala, and improved social processing	Not addressed	Hadjikhani et al. (2018)
Autism	Single center, placebo controlled, double blind (*n* = 92)	Children 7–15	2 mg	Long-term administration	Oral	1 mg twice daily	An effect of bumetanide was found on one of the Repetitive Behavior Scale-Revised	Diuretic effects, hypokalemia	Sprengers et al. (2021)
Schizophrenia	Double-blind, placebo controlled, human (*n* = 26)	55.9 ± 13.9 years, M/F	2 mg	Long-term administration	Oral	1 mg twice daily for 2 months	No difference between bumetanide or placebo on positive and negative PANSS or BPRS scores	None reported	Rahmanzadeh et al. (2017a)
Schizophrenia	Double-blind, placebo controlled, human (*n* = 24)	38–67, M/F	2 mg	Long-term administration	Oral	1 mg twice daily for 2 months	Improvement following 2 months of treatment assessed by PANSS	Electrolyte testing showed no adverse effects	Rahmanzadeh et al. (2017b)
Parkinson’s disease	Open label study (*n* = 4)	M/F > 50 years	5 mg	Long-term administration	Oral	5.0 mg once daily for 2 months	Improvements of PD motor symptoms in four patients, improvement of gait and freezing in two patients	Polyuria, but overall well tolerated	Damier et al. (2016)
Temporal lobe epilepsy	Case-study (*n* = 3)	Adult-31, 32, and 37 years, M	2 mg/day	Long-term administration	Oral	3 or 4 months + pre-existing anti-epileptic drugs, followed by 3-month posttreatment follow-up	Seizure frequency decreased; epileptiform discharges decreased on pre- vs post EEG	None reported	Eftekhari et al. (2013)

### Autism

A group of three studies showed that bumetanide treatment improved behavioral deficits at drug doses of 1–4 mg daily for 3 months in children and adolescent patients based on Childhood Autism Rating Scales (CARS) assessments (Table 3) (Lemonnier and Ben Ari, 2010; Lemonnier et al., 2012; Lemonnier et al., 2017). These doses correspond to approximately 0.028–0.08 mg/kg for children and 0.016–0.064 mg/kg bumetanide for adolescents. Another study using 1 mg bumetanide per day for 3 months in children ages 2–6 years old (0.05–0.08 mg/kg) resulted in no significant improvement based on the Autism Behavior Checklist (ABC) (Du et al., 2015) (Table 3). However, autism is most likely a disease with heterogenous etiologies, and evaluation of drug actions should be conducted based on patient stratification (Sprengers et al., 2021).

In autistic patients of an older age range of 14–28 years bumetanide (1 mg daily, 0.013–0.02 mg/kg) given for 10 months reduced amygdala activation induced by constrained eye contact and improved social processing (Hadjikhani et al., 2018) (Table 3). Further, a study of autism in patients age 7–15 years showed that bumetanide (2 mg/day, 0.037–0.089 mg/kg) improved behavior assessed by the Repetitive Behavior Scale, but not other behaviors assessed by the Sensory Profile and the Aberrant Behavior Checklist (Sprengers et al., 2021) (Table 3).

These bumetanide doses of 0.013–0.09 mg/kg are predicted to result in low brain levels of bumetanide below those that could inhibit NKCC1 based on rodent PK studies of this drug (Table 1). Thus, unless the PK-PD is significantly different between rodents and humans, it is unlikely that NKCC1 mechanisms are involved in bumetanide mechanisms to improve autism.

### Schizophrenia

In a double-blind placebo controlled randomized clinical trial of patients with schizophrenia aged 38–67 years, treatment with bumetanide (1 mg twice a day, 0.027 mg/kg) for 2 months resulted in improved behavior assessed by Positive and Negative Syndrome Scale (PANSS) scores (Rahmanzadeh et al., 2017b) (Table 3). The authors reported no considerable adverse events and discontinuations were equal in the two treatment arms (Rahmanzadeh et al., 2017b). A second double-blind randomized trial with the same group of 26 schizophrenia patients used the same dose of 1 mg BID (twice daily) and did not achieve any significant benefits on the PANSS or the Brief Psychiatric Rating Scale (BPRS) (Rahmanzadeh et al., 2017a). The low bumetanide dose of 0.027 mg/kg is again estimated to achieve brain levels of the drug below those effective for inhibition of NKCC1.

### Parkinson’s disease

In a small open label case series, four patients with Parkinsons’ disease (mean age 61 years) were titrated to daily doses of 5 mg once daily for several months with some suggestion of treatment benefit in motor scores and a putative effect on striatal GABAergic cells. The authors noted that excessive diuresis might preclude higher doses, however, no details of adverse events were provided. With doses of bumetanide (2 mg/day, 0.027 mg/kg) for 3 months the drug significantly improved motor function assessed by the stand-walk-sit (SWS) test and gait function assessed by Giladi’s questionnaire (Damier et al., 2016) (Table 3). This low bumetanide dose again implicates non-NKCC1 brain mechanisms.

### Epilepsy

In a study of adult epilepsy, patients were administered bumetanide at 2 mg/day (0.027 mg/kg) for 3 or 4 months which resulted in decreased seizure frequency (Eftekhari et al., 2013) (Table 3). These bumetanide doses may be predicted to yield low brain levels of this drug based on the rodent PK studies of bumetanide (Table 1), suggesting non-NKCC1 mechanisms. Interestingly, diuretics are known to have a therapeutic action in epilepsy (Hesdorffer et al., 2001).

### Clinical assessment of adverse effects of bumetanide

About half of these clinical studies reported no adverse effects (Lemonnier and Ben-Ari, 2010; Eftekhari et al., 2013; Du et al., 2015; Rahmanzadeh et al., 2017b) (Table 3). The other studies reported that bumetanide increased urine output (Kahle et al., 2009), hypokalemia (Lemonnier et al., 2012; Lemonnier et al., 2017; Sprengers et al., 2021) which was treated with supplemental potassium (Lemonnier et al., 2012; 2017), and orthostatic hypotension due to diuresis (Sprengers et al., 2021). These studies show that further reporting of adverse events with use of bumetanide across dose ranges are still needed for this medication to be taken into larger scale clinical trials.

## Clinical bumetanide doses regulate kidney diuresis through the NKCC2 transporter

### Bumetanide doses and PK parameters for diuresis in adults

**FIGURE 5:**
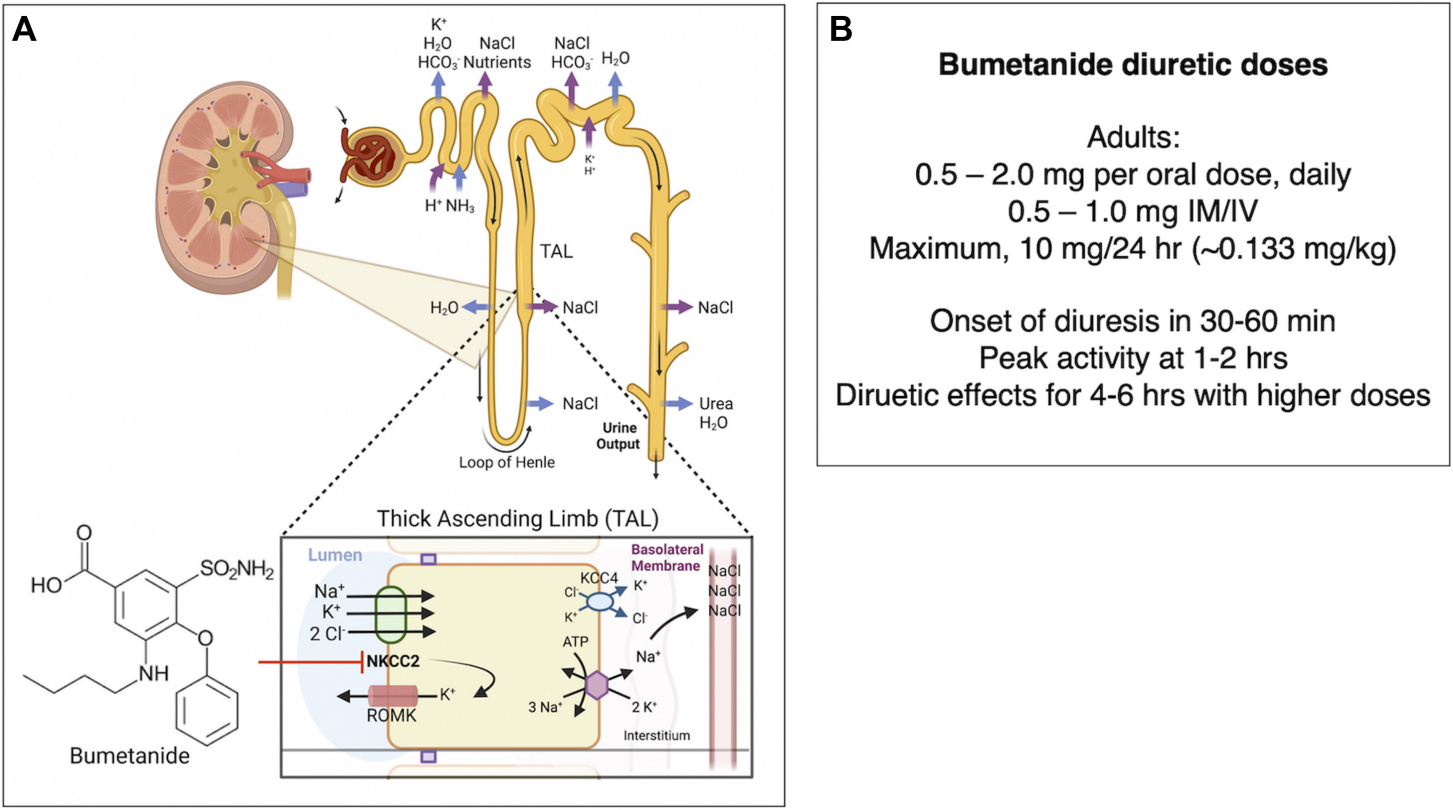
**(A)** Bumetanide regulation of diuresis by functional kidney regulation. Bumetanide inhibition of NKCC2 for diuresis. Bumetanide inhibits the NKCC2 sodium-potassium-chloride cotransporter located on the apical side of the thick ascending limb (TAL) of the loop of Henle. NKCC2 is responsible for Cl^−^ reabsorption from the lumen back to blood glomerular capillaries (Ares et al., 2011). These ions are transported from the TAL cells to the interstitial space, utilizing the electrochemical gradient generated by Na^+^/K^+^ ATPase (Burg, 1976). A K^+^-Cl^−^ cotransporter 4 (KCC4) facilitates transport of K^+^ and Cl^−^ into the interstitial space as well. Some K^+^ moves back into the lumen via the apical ROMK (Renal Outer Medullary K) channel (Mount, 2014). The K^+^ movement back into the lumen is essential to ensure that there is enough K^+^ in the lumen so that NKCC2 can function (Giebisch, 1993; Wang, 2006). Inhibition of NKCC2 by bumetanide leads to decreased reabsorption of Na^+^, K^+^, and Cl^−^, which leads to secretion of water due to the osmotic effect and increased diuresis (Hebert et al., 1984; Hebert and Andreoli, 1986). Bumetanide therapeutic response to diuresis includes decreased edema, reduced blood pressure through vasodilation, and decreased workload on the heart (Blaine et al., 2015). **(B)** Bumetanide doses for diuresis. Doses of bumetanide in adults for diuresis and time-frame of diuresis.

Bumetanide (Brand name Bumex) is an FDA-approved diuretic drug that antagonizes the kidney NKCC2 sodium-potassium-chloride cotransporter. The drug produces a rapid diuretic effect to increase urinary excretion of sodium chloride, and other electrolytes, acting at the ascending limb of the loop of Henle of the kidney (Figure 5) (Erker et al., 2016) as indicated by the FDA bumetanide label. Bumetanide inhibits sodium reabsorption in a dose-dependent manner.

In adults, bumetanide is usually given as an 0.5–2.0 mg/oral dose (PO) once daily-BID, or at 0.5–1.0 mg dose intramuscularly or intravenously (IM/IV). The maximal dose (PO/IM/IV) is normally 10 mg/24 h. Lower doses are given to infants and children (0.015 mg/kg to 0.1 mg/kg/dose once daily). Following oral dosing in adults, diuresis occurs in 30–60 min, peaks at 1–2 h, and is completed within 4 h. The pharmacokinetics of bumetanide by IV, IM, and PO routes display a biexponential equation including an initial disposition phase (t_1/2_, alpha = 5.1 min), followed by a slower elimination phase (t_1/2_, beta = 44 min). Bumetanide is almost completely absorbed (80%), and absorption is not altered when taken with food. The drug’s half-life times for IV, IM, PO solution, and PO tablet administration ranges from 24–86 min, 47–139 min, 27–71 min, and 26–99 min, respectively. Plasma protein-binding is in the range of 94%–96% (FDA Bumetanide Label, 2009). Approximately 70% of parenteral and 60% of oral bumetanide are excreted as intact drug in urine by 24 h after administration (Holazo et al., 1984). Urinary and biliary metabolites are formed by oxidation of the N-butyl side chain; metabolites have been found to lack significant diuretic effects (Löscher and Kaila, 2022).

### Geriatric patients and bumetanide

Use of bumetanide in the elderly, including AD patients, involves a lower rate of drug clearance compared to adults of moderate ages. In geriatric patients (ages 65–73), bumetanide clearance appears to be lower (1.8 ± 0.3 mL/min-kg) compared to younger subjects (2.9 ± 0.2 mL/min-kg) after a single 0.5 mg oral bumetanide dose. Maximum plasma concentrations are also higher in geriatric subjects (16.9 ± 1.8 ng/mL) compared to younger subjects (10.3 ± 1.5 ng/mL). Urine flow rate and total excretion following bumetanide administration increase at a lower rate compared to younger subjects (FDA Bumetanide Label, 2009).

## Strategies to enhance bumetanide bioavailability in the brain

### Clinical bumetanide doses provide efficacy to improve patient neurological deficits

**FIGURE 6:**
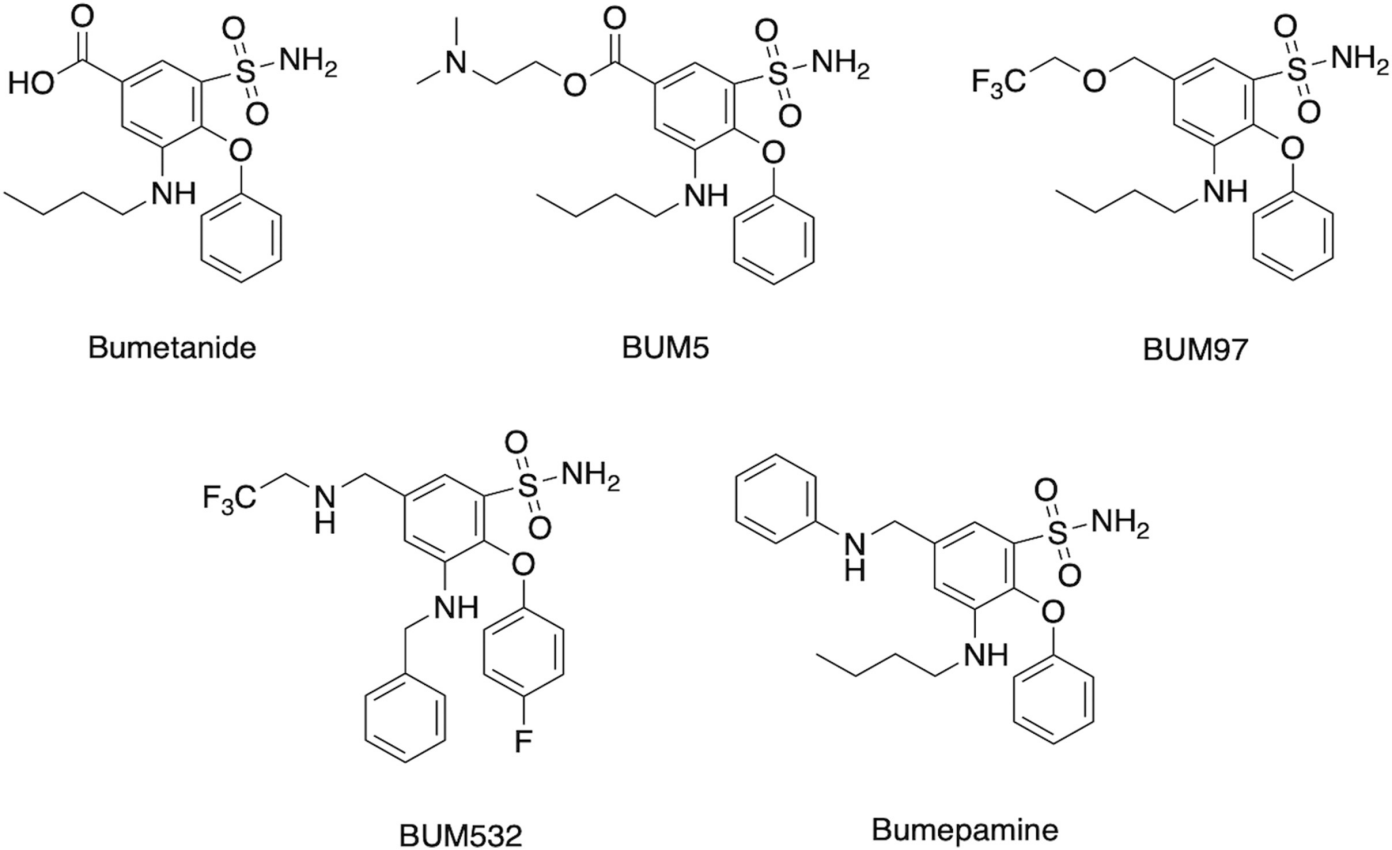
Bumetanide, prodrug, and derivatives. Structures are shown for bumetanide, the bumetanide derivatives of BUM97 and BUM 532 (Auer et al., 2020), the prodrug BUM5 (Erker et al., 2016), and the related bumepamine (Brandt et al., 2018).

Assessment of several clinical studies show that bumetanide administered at 1–4 mg per day can improve several neurological deficits at low brain drug levels likely through non-NKCC1 mechanisms. These doses of 1–4 mg/day are comparable to those used for bumetanide’s clinical use in diuresis for treatment of edema and hypertension-related disease conditions (explained in next section). Therefore, improvement of bumetanide efficacy for treating neurologic disorders may be achieved by increasing bioavailability to the brain. Enhanced brain penetrance may also be advantageous by reducing peripheral drug levels and unwanted diuresis for treatment of brain disorders. Enhancement of bumetanide brain levels include inhibition of drug transporters, combined with prodrug forms and derivatives of bumetanide (Figure 6).

### Inhibition of organic anion transporters

The poor brain bioavailability of bumetanide may be due to its active efflux by the organic anion transporters (OAT3, Oatp1-4) and MRP4 at the blood-brain barrier (BBB) (Romermann et al., 2017). Because 98%–99% of plasma bumetanide is protein-bound, only 1%–2% is available for passive penetration across the BBB (Cohen et al., 1976; Puskarjov et al., 2014; Topfer et al., 2014). Thus, inhibition of OATs may provide a strategy to improve brain bioavailability of the low-level unbound bumetanide in the periphery (Donovan et al., 2015). Co-administration of probenecid, an OAT3 inhibitor, significantly increases bumetanide levels in both the hippocampal extracellular fluid (ECF) and plasma in rats (Donovan et al., 2015). It would be interesting to assess co-administration of OAT inhibitors as an approach to increase brain levels of bumetanide in animal models of AD and other neurological deficits.

### Prodrug forms of bumetanide

Brain levels have been demonstrated to be improved by chemical analogs of bumetanide as prodrugs (Erker et al., 2016; Savardi et al., 2021; Theilmann et al., 2021; Löscher and Kaila, 2022). Lipophilic prodrug analogs and related variant molecular entities have been studied to overcome the poor BBB penetrance of bumetanide. One example is BUM5 (N,N-dimethylaminoethylester, also termed DIMAEB or STS5) (Figure 6) which displays 3.5-fold higher brain levels compared to bumetanide after systemic administration, and less diuretic effects than bumetanide since it is metabolized to the active drug form in the brain (Tollner et al., 2014; Erker et al., 2016). Follow-up studies assessed co-administration of BUM5 with a GABA-mimetic phenobarbital (PB) which resulted in increased potentiation of anti-epileptic effects, (Erker et al., 2016). The rationale for ester prodrugs such as BUM5 is that the high blood flow per unit area in brain enables small, lipophilic prodrugs to penetrate the parenchyma rapidly and convert to the parent drug via esterases. In support of this approach, BUM5 has been reported to improve ischemic infarction, swelling, and neurological deficits (Huang et al., 2019).

### Bumetanide derivatives

Other lipophilic bumetanide derivatives have been evaluated in *in vivo* models, both singly and in combination with phenobarbital (PB) (Auer et al., 2020). Several R-group modifications including allyl to trifluoro-ethyl exchange (BUM97), nitrogen for oxygen in the trifluoro-ethyl group (BUM532), and allyl group exchange for the trifluoroethyl group (BUM532) were achieved to compare efficacy and penetrance (Figure 6). Of the derivatives tested, BUM97 showed suppression of epileptiform activity and synergistic anticonvulsant effects with sub-effective dose of PB, while BUM532 in combination with a sub-effective dose of PB showed increased seizure thresholds.

Novel bumetanide analogs with various substitutions in the R1, R3, and R5 have been tested using a ligand-based computational strategy model (Savardi et al., 2020). The carboxylic acid moiety in the R1 position was essential for blocking NKCC1 *in vitro*. Further, modifications of the linear alkyl chain in R3 and demethylation of the sulfonamide in R5 was reported to decrease drug potencies against NKCC2, while retaining NKCC1 potency (Savardi et al., 2020). This kind of increase in selectivity has been contested (Löscher and Kaila, 2022), and the field lacks a drug that would be effective in selectively blocking NKCC1. Moreover, the recent data (Kurki et al., 2022) on NKCC1a and NKCC1b expression profiles (non-neuronal and neuronal, respectively) raise more challenges in attempts to specifically block NKCC1-mediated Cl^−^ uptake in neurons.

Of interest is a non-acidic bumetanide derivative called bumepamine that lacks NKCC1 and NKCC2 effects but shows efficacy in alleviating brain dysfunctions. In a birth asphyxia rat model, bumepamine was more effective than bumetanide and DIMAEB in potentiating the anticonvulsant effects of phenobarbital (Johne et al., 2021a; Johne et al., 2021b). Bumepamine did not suppress network events driven by depolarizing GABA in neonatal hippocampal slices, nor did it inhibit Ca^2+^ transients induced by depolarizing GABA_A_-receptor currents in hippocampal slices since it lacks direct inhibitory action on NKCC1 (Brandt et al., 2018). Brain levels of bumepamine were ∼7-fold higher than bumetanide at equimolar peripheral doses (Brandt et al., 2018; Löscher and Kaila, 2022) and bumepamine displayed lower plasma levels compared to bumetanide. These findings provide support for non-NKCC1 mechanisms for bumepamine and related drugs in the amelioration of brain dysfunctions.

## Bumetanide toxicity to be considered in AD

Long-term use of bumetanide for chronic brain dysfunctions including AD should consider effective drug doses that improve the neurological disorder with tolerable diuresis effects. The effective bumetanide doses utilized in clinical trials of neurological diseases are in the range used for diuresis (Table 3) and, thus, situations of long-term diuretic treatment by bumetanide suggest that the diuresis may be tolerable and manageable during lengthy treatment for AD. Long-term hypokalemia may be managed by potassium supplements.

In geriatric AD patients, adverse bumetanide effects may include compromised circulatory function involving hemodynamic instability (FDA bumetanide label, 2009). Further side effects consist of muscle cramps, dizziness, hypotension, headache, nausea, thrombocytopenia, and deviations in complete blood cell (CBC) counts (0.1%) (FDA bumetanide label, 2009). Loop diuretics such as bumetanide can induce temporary hearing loss, but might cause more severe damage if applied in conditions of severe acute or chronic renal failure along with other ototoxic drugs (such as aminoglycoside antibiotics, NSAIDS, quinine, and acetaminophen) (Ding et al., 2016; Joo et al., 2018). Bumetanide-induced ringing in the ears or hearing loss likely occurs through alteration of cochlear activity in a manner similar to other loop diuretics such as furosemide and ethacrynic acid (Quick and Duvall, 1970; Brown, 1976).

## Patient exposure to bumetanide decreases the incidence of AD

Analysis of patient EHR records show that exposure to bumetanide, at clinical doses routinely utilized for diuresis, results in substantial decreases in the incidence of AD in individuals over 65 years of age in cross-sectional single time-point analyses (Taubes et al., 2021). Two independent EHR databases have beeen analyzed. One EHR database consists of the University of California at San Francisco (UCSF) containing medical records of 1.3 million patients, of which 1,850 patients (57.2% men and 42.8% women) over the age of 65 years had used bumetanide. The other EHR resource was from the Mount Sinai Health System (MSHS) that covers 3.9 million patients among five hospitals in the New York City area; among these patients, 2,848 individuals (50.2% men and 49.8% women) over the age of 65 years had used bumetanide. Since bumetanide is typically prescribed for hypertension or edema, control and bumetanide cohorts included scores for those with hypertension and edema combined with age, sex, and race. These data were calculated for AD prevalence as the ratio of patients with AD to total patients; the analysis illustrated a significant and substantial reduction in AD prevalence of 35%–75% in bumetanide-treated individuals compared to controls (Taubes et al., 2021). Furthermore, another control group of patients exposed to non-loop diuretics showed a reduction by 40%–70% of AD prevalence in the bumetanide group compared to the non-loop diuretic group. It is noted, however, the APOE4 genotype of these groups of patients was unknown and further studies to assess EHR data stratified for the APOE4 genotype will be desirable. Overall, these findings provide evidence that bumetanide may effectively reduce the onset of AD in the aged population (65 years and older).

Importantly, the beneficial outcomes of bumetanide in reducing the incidence of AD occurred at clinical doses routinely used for treatment of hypertension and edema. These general doses of 0.5–2 mg per day, with a maximum of 10 mg per day, result in diuresis with manageable side effects. Therefore, assessments of bumetanide at routine clinical doses for reducing the incidence of AD will be of great importance in future studies.

## Summary and conclusion: evidence supports repurposing bumetanide as a candidate AD therapeutic

The clinical electronic health records (EHR) analyses suggest that bumetanide acts at the early stages of AD to prevent progression to AD deficits of cognitive impairment. These clinical data further suggest that bumetanide can influence AD progression at doses utilized for diuresis for which safety and toxicity properties of the drug are known. Broader assessment of patient health records will be important to provide further evidence of the hypothesized beneficial association of bumetanide treatment for reduced AD incidence and progression in the APOE4 stratified population of AD.

Bumetanide’s mechanism of action to improve brain dysfunction has been discussed as occurring by antagonism of the NKCC1 transporter to promote hyperpolarizing GABAergic neuronal signaling as observed in the healthy brain. However, improvement of memory deficits in APOE4 mice and beneficial effects for other brain dysfunctions show that effective bumetanide brain levels in the nM range are below the level of 100–300 nM required for NKCC1 inhibition (Table 2). These data support the hypothesis for non-NKCC1 mechanisms within the brain and/or peripheral effects by bumetanide to improve memory deficits in the APOE4 stratified risk factor condition. Future experiments to address the above mechanism may utilize NKCC1 gene knockout in the APOE4 mice for bumetanide testing; bumetanide efficacy in mice lacking NKCC1 would indicate a non-NKCC1 mechanism. It will also be of interest to conduct pharmacological assessment of bumetanide dose-response relationships for rescuing memory deficits in APOE4 mice.

Studies of bumetanide pharmacodynamics are needed to identify biomarkers related to drug efficacy for ameliorating memory deficits in APOE4-related mouse models of AD. Bumetanide rescues AD-like excitability and LTP deficits in hippocampal brain slices from APOE4 mice (Taubes et al., 2021). Neuronal excitability is the fundamental regulator of activity-dependent neurotransmitter release for cell-cell communication among brain neural circuits (Brady et al., 2011) and, thus, it is logical to hypothesize that changes in deficits of synaptic neurotransmitter components occur in AD that may be ameliorated by bumetanide. Assessment of neurotransmitter components, consisting of the major group of neuropeptides and classical small molecule transmitters, is readily achieved by peptidomics and metabolomics approaches to define neurotransmitter signatures (Hook and Bandeira, 2015; Hook et al., 2019; Podvin et al., 2022) and can be planned for future studies. Since bumetanide reduces amyloid plaque load in the J20-APOE4 mouse model of AD, it will be fruitful to assess Aβ peptide forms as possible biomarkers of bumetanide efficacy.

Efforts to increase the bioavailability of bumetanide and/or its analogs in the brain are worth investigating. The possibility of prodrug formulation, inhibition of efflux mechanisms, and other efforts to enhance brain effects while reducing the diuretic peripheral kidney effects are desirable, particularly in the largely geriatric target population of AD.

In conclusion, the efficacy of bumetanide to improve memory deficits in the APOE4 genetic condition of AD and evidence for its clinical effectiveness to reduce the incidence of AD provide compelling support for further clinical investigation of bumetanide as a repurposed therapeutic for ameliorating AD deficits.
